# Exogenous Application of Selenium Nanoparticles Boosted the Drought Tolerance in Wheat by Increasing Morpho-Physiochemical and Yield Attributes

**DOI:** 10.1155/sci5/7789912

**Published:** 2025-10-30

**Authors:** Shams Shaila Islam, Md. Mozammal Haque, Md. Asikuzzaman Sohag, Syed Nazmul Haque, Md. Rayhanul Hoque, Bikash Chandra Sarker, Ahmed Khairul Hasan, Rashed Karim, Thanet Khomphet

**Affiliations:** ^1^Department of Agronomy, Hajee Mohammad Danesh Science and Technology University, Dinajpur 5200, Bangladesh; ^2^Department of Soil Science, Hajee Mohammad Danesh Science and Technology University, Dinajpur 5200, Bangladesh; ^3^Department of Agricultural Chemistry, Hajee Mohammad Danesh Science and Technology University, Dinajpur 5200, Bangladesh; ^4^Department of Agronomy, Bangladesh Agricultural University, Mymensingh 2202, Bangladesh; ^5^Department of Geography, New Degree Government College, Rajshahi 6200, Bangladesh; ^6^School of Agricultural Technology and Food Industry, Walailak University, Nakhon Si Thammarat 80160, Thailand; ^7^Herbology Research Center, Walailak University, Nakhon Si Thammarat 80160, Thailand

**Keywords:** drought stress, foliar application, selenium nanoparticles, yield

## Abstract

The present study aimed to determine whether sole or joint Se treatments improve wheat growth under drought conditions. The study was conducted using two wheat varieties, BARI Gom30 and BARI Gom33, at the Agronomy Research Field of Hajee Mohammad Danesh Science and Technology University, Dinajpur, during the Rabi season from December 2023 to April 2024. A randomized complete block design with three replications was employed. Wheat plants were grown under drought conditions in the field, and seedlings in each plot were subjected to six treatments: T_0_ (control, no selenium), T_1_ (10 ppm Se), T_2_ (20 ppm Se), T_3_ (30 ppm Se), T_4_ (40 ppm Se), and T_5_ (50 ppm Se) per plot. The results indicated that the highest plant height (103.35 cm), number of leaves per plant (5.50), number of tillers per plant (5.00), leaf area (5125.95 mm^2^), leaf temperature (26.53°C), spike length (17.75 cm), and biological yield (4.33 t ha^−1^) were observed in the T5 treatment (50 ppm Se per plot). However, the maximum grain yield was obtained with T_3_ (30 ppm Se). The highest harvest index was recorded in the T_4_ treatment (40 ppm Se), suggesting its effectiveness in optimizing yield distribution. Additionally, physiological parameters such as chlorophyll content, carotenoid levels, relative water content, water saturation deficit, water retention capacity, water use efficiency, leaf succulence, excised leaf water loss, and cell membrane stability showed slightly higher peak values in BARI Gom33 (V2), indicating its superior drought tolerance and yield potential. Based on the findings, a Se concentration of 50 ppm (T5 × V2) was identified during the interaction as the most effective treatment for enhancing the growth of the selected wheat varieties (V2) under drought conditions.

## 1. Introduction

Wheat (*Triticum aestivum* L.) is one of the most widely cultivated cereal crops globally, serving as a staple food for billions of people. In 2020, total global wheat production reached approximately 760 million metric tons of wheat while grain supplying nearly 20% of the total caloric and protein intake for approximately 4.5 billion people worldwide [1, 2]. In Bangladesh, wheat production increased sixfold between 1961 and 2017, from 5000 to 30,000 metric tons. However, according to the Food and Agriculture Organization [3], temperature anomalies have adversely affected wheat productivity, raising concerns about future food security. As a result, current wheat production in Bangladesh has declined to approximately 20,000 metric tons.

Temperature fluctuations leading to drought play a critical role in determining optimal wheat growth and productivity [4]. Water deficiency adversely affects various physiological and metabolic processes in wheat and is considered a major yield-limiting factor. This presents a significant challenge for achieving higher crop yields while simultaneously conserving and efficiently utilizing depleting underground water resources. One potential strategy to address this issue is the induction of drought tolerance in wheat, which has become a focal point of research due to the increasing scarcity of water resources.

Drought stress represents a major threat to global agricultural productivity, a challenge increasingly intensified by dwindling water resources and the accelerating impacts of climate change [5]. The response of wheat plants to drought is highly complex, and the severity of drought can influence plant reactions to other environmental stresses. The effects of drought stress on plants have not been properly described. Drought stress primarily leads to cellular impairments, including osmotic stress, dehydration, dysfunction of endosomal and plasma membranes, loss of turgor pressure, depletion of energy, inhibition of metabolite synthesis, oxidative stress, nutrient imbalance, and impaired photosynthesis [5]. Drought and heat stress, often occurring together, severely limit small-grain cereal yields—especially during terminal growth stages in wheat across arid and semiarid regions [6]. Consequently, mitigating drought-induced stress in wheat production has become a global priority.

Selenium is a trace mineral essential for various biological functions. The recommended daily Se intake ranges from 25 to 34 μg for adults and 6–22 μg for children. As a potent antioxidant, Se helps combat oxidative stress and protects against chronic diseases such as cancer and cardiovascular disorders. Similarly, in plants, Se functions as an antioxidant at lower concentrations, enhancing resistance to environmental stressors, including drought and high temperatures [7]. Studies have shown that low Se concentrations can protect wheat seedlings from cold stress [8], mitigate high-temperature effects in sorghum, enhance drought tolerance in rapeseed (*Brassica napus* L.) seedlings [9], and prevent desiccation in silver maple (*Acer saccharinum* L.) [10]. Under drought stress conditions, the application of 3 mg L^−1^ Se improved branching in okra (*Abelmoschus esculentus* L.) [11]. Moreover, Se has been reported to enhance membrane stability and relative water content in wheat and strawberries (*Fragaria* × *ananassa*) [12]. In sesame, foliar application of Se under drought stress increased proline accumulation in leaves [13], while soil application enhanced peroxidase enzyme activity [14].

Selenium fertilization has been shown to improve wheat productivity under drought conditions by increasing the number of productive tillers, grains per spike, and overall grain yield [15]. Beyond bulk Se applications, selenium nanoparticles (SeNPs) have also been demonstrated to enhance crop tolerance to abiotic stress. For example, applying 30 mg L^−1^ SeNPs to wheat seedlings under drought conditions significantly improved plant height and shoot length [16]. These findings suggest that direct Se application may mitigate the adverse effects of drought stress. Given the antioxidant properties of Se, we hypothesize that foliar application of Se can enhance wheat grain yield by improving drought tolerance. Therefore, the main objective of this study is to determine whether sole or joint Se treatments improve wheat growth under drought conditions.

## 2. Materials and Methods

### 2.1. Experimental Setup and Plant Material

This study was conducted on two wheat varieties, BARI Gom30 (V_1_) and BARI Gom33 (V_2_), at the Agronomy Research Field of the Department of Agronomy, Hajee Mohammad Danesh Science and Technology University, Dinajpur, during the Rabi season from December 2023 to April 2024. The experiment was designed as a randomized complete block design (RCBD) with three replications. Wheat plants were cultivated under natural field conditions. Each experimental plot measured 4 × 2.5 m (10 m^2^), with ten plots in total. Seeds were sown at a uniform depth in each plot. One week after germination, only three uniformly growing seedlings per hill were retained for further study. Fertilizers were applied at regular intervals (every 8–12 days). Soil moisture stress was introduced when the seedlings reached 20 days of age.

The seedlings were divided into three groups based on soil moisture levels: (1) control—grown under normal water conditions at field capacity (12.5 ± 0.05% soil saturation), (2) moderate moisture stress—grown under moderate soil moisture stress (8.5 ± 0.05% soil saturation), and (3) severe moisture stress—subjected to severe soil moisture stress (4.5 ± 0.05% soil saturation). Tensiometers were installed in every plot to monitor the level of moisture in the soil. To maintain the designated soil moisture levels, daily watering was adjusted based on plot weight. All plants received equal amounts of fertilizer solution, while water application varied to meet their respective soil moisture conditions. Each soil moisture level was further divided into six selenium (Se) treatments: T_0_ (control): no selenium application, T_1_: 10 ppm Se (10 mg L^−1^), T_2_: 20 ppm Se (20 mg L^−1^), T_3_: 30 ppm Se (30 mg L^−1^), T_4_: 40 ppm Se (40 mg L^−1^), and T_5_: 50 ppm Se (50 mg L^−1^). Sodium selenite (Na_2_SeO_3_) was dissolved in water and applied as a foliar spray at three growth stages: vegetative stage, preflowering stage, and maturity stage.

The experiment was conducted under a manually operated rainout shelter equipped with a flexible, translucent plastic sheet to prevent unintended rainfall exposure. Leaf samples from all Se treatments and soil moisture levels (control, moderate, and severe moisture stress) were collected at different growth stages from three replications. Data were recorded from ten randomly selected plants per plot for further analysis.

### 2.2. Morphological Traits

Morphological traits like plant height, leaf number plant^−1^, tiller number plant^−1^, leaf area, leaf temperature, spike length, biological yield, yield, and harvest index were measured.

### 2.3. Morpho-Physiological Traits

#### 2.3.1. Photosynthetic Pigments

The chlorophyll content of wheat leaves in each treatment was determined following the method outlined by Talukder et al. [17]. The leaves weighing 0.10 g were carefully taken and their weight was recorded. The leaves were submerged in 10 mL of acetone at room temperature for 48 h in a dark environment. Subsequently, the sample underwent agitation on an electric horizontal shaker overnight. For measuring chlorophyll, chlorophyll (a, b), total chlorophyll content, and carotenoid, the optical density or absorbance of the supernatant was recorded using a UV-visible spectrophotometer at wavelengths of 663, 645, and 470 mm. The concentration of chl a, chl b, and total chlorophyll was measured using the following formula.

(1) $$\begin{array}{c} \text{Chlorophyll }a\;\left(\text{mg }g^{-1}\text{ leaf}\right)=\frac{\left[12.7\left(D_{663}\right)-2.69\left(D_{645}\right)\right]\times V}{1000\times W},\\ \text{Chlorophyll }b\;\left(\text{mg }g^{-1}\text{ leaf}\right)=\frac{\left[22.9\left(D_{645}\right)-4.68\left(D_{663}\right)\right]\times V}{1000\times W},\\ \text{Total chlorophyll }\left(\text{mg }g^{-1}\text{ leaf}\right)=\frac{\left[20.9\left(D_{470}\right)+8.02\left(D_{663}\right)\right]\times V}{1000\times W},\\ \text{Carotenoid }\left(\text{mg }g^{-1}\text{ leaf}\right)=0.216\times D_{663}–0.304\times \left.D_{645}\right)\times 0.452\times D_{470}, \end{array}$$

where *D* = absorbance reading of the chlorophyll extract at the specific wavelength, *V* = final volume of the 80% acetone – chlorophyll extract, and *W* = fresh weight in gram of the tissue extracted.

#### 2.3.2. Cell Membrane Stability (CMS)

Leaf samples (1 g) were collected, rinsed with distilled water to eliminate any attached electrolytes, and placed in vials containing 10 mL of distilled water in order to measure the stability of the cell membrane. After being sealed in the vials for 6 hours at 25°C, the electrical conductivity of the solution (EC_1_) was measured. Following this, samples were subjected to an electric oven at 90°C for 2 hours, and the electrical conductivity (EC_2_) of solution was estimated. CMS was measured in percentage as follows:

(2) $$\begin{array}{c} \text{CMS }\left(\%\right)={\frac{{\text{EC}}_{1}}{{\text{EC}}_{2}}}_{1}\times 100, \end{array}$$

where EC_1_ = electrical conductivity measured at 25°C test tubes and EC_2_ = electrical conductivity measured at 90°C test tubes.

### 2.4. Water Status (%)

Leaf chlorophyll contents (LCCs) and stomatal conductance (SC) were recorded using a chlorophyll meter (model CL-01 Hansatech Instruments Ltd., United Kingdom) and an automatic porometer MK-3 (Delta-T Devices, Burwell Cambridge, England), respectively. Leaf relative water contents (LRWCs) were determined using fully expanded young leaves. Fresh wheat leaves were immediately shifted to the laboratory to measure fresh weight (FW) and then leaves were soaked in distilled water for 12 h at room temperature to record leaf turgid weight (TW). Then the leaves were oven dried at 70°C till constant weight to find out the dry weight (DW) of leaves. LRWC was calculated by the following formula.

(3) $$\begin{array}{c} \text{LRWC }\left(\%\right)=\text{FW}-\frac{\text{DW}}{\text{TW}}-\text{DW}\times 100. \end{array}$$

Leaf water potential (LWP), leaf osmotic potential (LOP), and leaf turgor potential (LTP) were determined by following the standard procedures/protocols described by Iqbal et al. [18]. Water use efficiency (WUE) was calculated by the given formula, WUE = grain yield/total water applied. Excised leaf water loss (ELWL) was measured by taking fully expanded young leaves (fourth from top) from each plant and then transferring them to the laboratory immediately. The leaves were weighed three times: immediately after harvest FW, after 6 h shade drying wilted weight (WW), and after oven drying at 70°C for 24 h DW. ELWL was then calculated by the formula given below [19].

(4) $$\begin{array}{c} \text{ELWL }\left(\%\right)=\text{FW}-\frac{\text{WW}}{\text{DW}},\\ \text{RWC}=\frac{\text{FW}-\text{DW}}{\text{TW}-\text{DW}}\times 100,\\ \text{WSD}=100-\text{RWC},\\ \text{WRC}=\frac{\text{TW}}{\text{DW}},\\ \text{WUC}=\frac{\text{TW}-\text{FW}}{\text{DW}}, \end{array}$$

where FW = fresh weight, WW = wilting weight, DW = dry weight, TW = turgid weight, WSD = water saturation deficit, ELWL = excised leaf water loss, RWC = relative water content, WRC = water retention capacity, and WUC = water use capacity.

## 3. Results and Discussion

### 3.1. Correlation of Wheat Morphological Traits With Different Levels of Se Application

**Figure 1:**
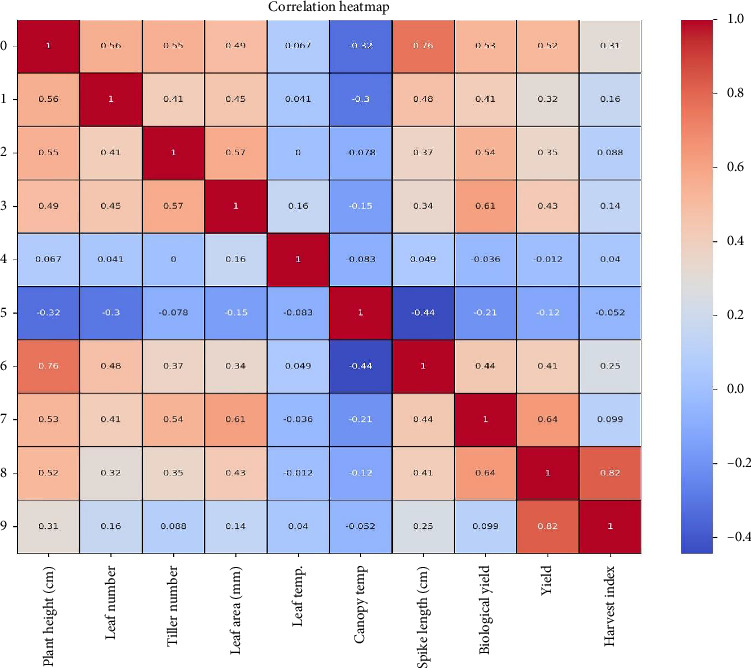
Combined correlation heatmap illustrating the relationships among various yield-contributing traits. The color scale on the right indicates the correlation strength and direction, where red denotes a strong positive correlation and blue represents weak or negative correlations.

The study revealed several significant relationships between various agronomic traits influencing wheat growth and yield (Figure 1). Plant height demonstrated a strong positive correlation with spike length (0.763^∗∗^), number of leaves (0.558^∗∗^), tiller number (0.553^∗∗^), biological yield (0.531^∗∗^), yield (0.517^∗∗^), and leaf area (0.495^∗∗^), indicating that taller plants tend to exhibit enhanced development in these aspects. However, it showed a weak but positive correlation with harvest index (0.311^NS^) and leaf temperature (0.067^NS^). No significant negative correlation was observed with canopy temperature (−0.323^NS^).

The number of leaves was positively and significantly correlated with spike length (0.478^∗∗^), leaf area (0.451^∗∗^), tiller number (0.412^∗^), and biological yield (0.409^∗^), suggesting its role in supporting plant growth and productivity. However, its correlation with yield (0.315^NS^) and harvest index (0.163^NS^) was weak and nonsignificant, and a negative but nonsignificant correlation was observed with canopy temperature (−0.297^NS^).

Tiller numbers showed strong positive correlations with leaf area (0.570^∗∗^), biological yield (0.542^∗∗^), number of leaves (0.412^∗^), spike length (0.371^∗^), and yield (0.354^∗^), emphasizing its contribution to wheat productivity. However, its correlation with harvest index (0.088^NS^) and leaf temperature (0.000^NS^) was negligible, with a nonsignificant negative correlation with canopy temperature (−0.078^NS^).

Leaf area exhibited significant positive correlations with biological yield (0.612^∗∗^), tiller number (0.570^∗∗^), number of leaves (0.451^∗∗^), yield (0.426^∗∗^), and spike length (0.345^∗^), indicating its influence on plant productivity. However, its correlation with leaf temperature (0.158^NS^) and harvest index (0.144^NS^) was weak and nonsignificant. Additionally, it showed a nonsignificant negative correlation with canopy temperature (−0.154 ^NS^), suggesting a limited role in temperature-related traits.

Leaf temperature displayed weak positive correlations with leaf area (0.158^NS^), spike length (0.049^NS^), number of leaves (0.041^NS^), harvest index (0.040^NS^), and tiller number (0.00 ^NS^), but none were statistically significant. It also showed nonsignificant negative correlations with canopy temperature (−0.083^NS^), biological yield (−0.036^NS^), and yield (−0.012^NS^), indicating its limited impact on these traits.

Canopy temperature was negatively correlated with spike length (−0.444^∗∗^), suggesting that higher canopy temperatures may hinder spike development. It also exhibited negative correlations with number of leaves (−0.297^NS^), biological yield (−0.207^NS^), leaf area (−0.154^NS^), yield (−0.120^NS^), leaf temperature (−0.083^NS^), tiller number (−0.078^NS^), and harvest index (−0.052^NS^), though most were not statistically significant.

Spike length had strong positive correlations with number of leaves (0.478^∗∗^), biological yield (0.441^∗∗^), yield (0.412^∗^), tiller number (0.371^∗^), and leaf area (0.345^∗^), indicating its association with improved growth and productivity. However, its correlation with harvest index (0.246^NS^) and leaf temperature (0.049^NS^) was weak and nonsignificant. It also exhibited a significant negative correlation with canopy temperature (−0.444^∗∗^).

Biological yield was significantly correlated with yield (0.636^∗∗^), leaf area (0.612^∗∗^), tiller number (0.542^∗∗^), spike length (0.441^∗∗^), and number of leaves (0.409^∗^), reinforcing its critical role in plant productivity. While it showed a positive but nonsignificant correlation with harvest index (0.099^NS^), it was negatively correlated with canopy temperature (−0.207^NS^) and leaf temperature (−0.036^NS^), though these correlations were not statistically significant.

Yield exhibited strong positive correlations with harvest index (0.823^∗∗^), biological yield (0.636^∗∗^), leaf area (0.426^∗∗^), spike length (0.412^∗^), and tiller number (0.354^∗^), highlighting the collective contribution of these traits to grain production. Its correlation with the number of leaves (0.315 ^NS^) was positive but nonsignificant.

Harvest index showed a strong positive correlation with yield (0.823^∗∗^) but exhibited weak and nonsignificant positive correlations with spike length (0.246^NS^), number of leaves (0.163^NS^), leaf area (0.144^NS^), biological yield (0.099^NS^), tiller number (0.088^NS^), and leaf temperature (0.040^NS^). It also had a nonsignificant negative correlation with canopy temperature (−0.052^NS^), suggesting that its primary reliance is on yield rather than other factors.

### 3.2. Plant Height

The wheat varieties exhibited a positive response to selenium application in terms of plant height, with statistically significant differences observed. Among the two varieties, BARI Gom33 (V_2_) attained the greatest plant height compared to BARI Gom30 (V_1_). Plant height varied significantly across different Se concentrations. The highest plant height was recorded in T_5_ (103.35 cm), whereas the lowest was observed in T_0_ (67.07 cm). The intermediate treatments yielded plant heights of 99.67 cm (T_1_), 98.27 cm (T_2_), and 99.75 cm (T_3_). The interaction effect between wheat variety and Se concentration was statistically significant (Table 1). The tallest plants were observed in T_5_ (50 ppm Se) applied to BARI Gom33 (V_2_), which was statistically comparable to T_4_ (40 ppm Se) applied to BARI Gom33 (V_2_). Conversely, the shortest plants were recorded in the T_0_ × V_1_ treatment, where no Se was applied to BARI Gom30 (Table 1). These findings align with previous research. The application of 30 mg·L^−1^ SeNPs to wheat seedlings under drought stress significantly enhanced plant height and shoot length [20], likely due to increased starch accumulation in chloroplasts and the protection of cellular content via enhanced secondary metabolite concentrations [21]. These findings are further supported by Naseem et al. [22] and Galic et al. [23], who also reported positive correlations between Se application and plant growth.

### 3.3. Leaf Number

The two wheat cultivars exhibited significant variations in vegetative growth parameters, particularly in leaf number. BARI Gom33 (V_2_) produced a higher number of leaves compared to BARI Gom30 (V_1_). Selenium treatments had a significant (*p* < 0.05) effect on the number of leaves in wheat. The highest leaf count (5.5 leaves) was observed in the T_5_ treatment (50 ppm Se), while the lowest (3.8 leaves) was recorded in T_4_, which was statistically similar to T_2_ (5.0 leaves). The interaction between wheat variety and Se concentration was also statistically significant (Table 1). The maximum number of leaves was recorded in T_5_ (50 ppm) × V_2_ (BARI Gom33), which was statistically comparable to T_1_ (10 ppm) × V_2_ (BARI Gom33) and T_5_ (50 ppm) × V_1_ (BARI Gom30). In contrast, the lowest leaf count was observed in the T_0_ × V_1_ (BARI Gom30) treatment, where no Se was applied (Table 1). Selenium application at low concentrations has been shown to enhance leaf development in wheat by strengthening antioxidant defenses, improving photosynthetic efficiency, and increasing stress tolerance. Yadav et al. [24] reported that foliar Se application improved physiological growth in wheat under both stressed and nonstressed conditions [25], particularly during stem elongation and flowering stages, which contributed to an increased number of leaves [26].

### 3.4. Tiller Number

Tiller number was significantly influenced by different Se concentrations in response to wheat variety. The highest tiller number (5.00 tillers) was recorded in T_5_ (50 ppm Se) for V_2_ (BARI Gom33), outperforming V_1_ (BARI Gom30), while the lowest tiller number (2.12 tillers) was observed in T_1_ (10 ppm Se). The interaction effect between wheat variety and Se concentration was statistically significant, with the highest tiller number observed in T_5_ (50 ppm) × V_2_ (BARI Gom33). This was statistically comparable to T_1_ (10 ppm) × V_2_ (BARI Gom33), T_4_ (40 ppm) × V_2_ (BARI Gom33), and T_5_ (50 ppm) × V_1_ (BARI Gom30). Conversely, the lowest tiller number was recorded in T_2_ (20 ppm) × V_2_ (BARI Gom33) (Table 1). Previous studies support these findings. Ikram et al. [27] reported that 30 mg L^−1^ of SeNPs effectively enhanced wheat growth under drought conditions. Sadak and Bakhoum [28] further highlighted that selenium improves plant water status by enhancing root water uptake capacity, leading to increased production under drought stress due to a higher number of productive tillers [29].

### 3.5. Leaf Area

Selenium significantly influenced drought tolerance in wheat by enhancing leaf area (Table 1). T_5_ (50 ppm Se) resulted in the largest leaf area (5125.95 mm^2^) in V_1_ (BARI Gom30), whereas the smallest leaf area (2133.65 mm^2^) was observed in T_0_ (control), followed by T_1_ (2582.22 mm^2^). There was no statistically significant difference between T_3_ (3397.84 mm^2^) and T_4_ (3444.54 mm^2^). In terms of interaction effects, the maximum leaf area was recorded in T_5_ (50 ppm Se) × V_2_ (BARI Gom33), which was statistically comparable to T_5_ (50 ppm Se) × V_1_ (BARI Gom30). Conversely, the minimum leaf area was observed in T_1_ (10 ppm Se) × V_1_ (BARI Gom30) (Table 1). These findings align with previous research. Rajala et al. [30] reported that drought stress reduces photosynthesis, plant water content, and leaf area development. Additionally, Akladious [31] found that selenium positively affects mesophyll cells, helping maintain membrane stability and permeability under drought conditions. Rady et al. [32] further confirmed that the application of Se25 or Se50 enhances wheat leaf surface area. At optimal levels, selenium promotes leaf expansion, acting as a strong carbohydrate sink, as demonstrated in lettuce [21].

### 3.6. Leaf and Canopy Temperature

There were insignificant differences among all the treatments of leaf and canopy temperature having zero interaction effects (Table 1).

### 3.7. Spike Length

Selenium had a significant impact on spike length, with BARI Gom33 (V_2_) producing longer spikes compared to BARI Gom30 (V_1_). Among the different Se concentrations, the T_5_ treatment (50 ppm Se) resulted in the longest spike length (17.55 cm), which was statistically similar to T_1_ (16.73 cm), T_2_ (16.85 cm), and T_3_ (16.92 cm). The T_0_ treatment (control) exhibited the shortest spike length (15.88 cm), followed by T_4_ (16.72 cm). Regarding the interaction effect between wheat varieties and Se concentrations, the longest spike length was observed in T_5_ (50 ppm Se) × V_2_ (BARI Gom33), which was statistically comparable to T_3_ (30 ppm Se) × V_2_ (BARI Gom33). The shortest spike length (4.96 cm) was recorded in T_0_ × V_1_ (BARI Gom30) (Table 1). These results align with previous studies. Fischer [33] reported that water stress during the flowering stage reduces kernel number per spike in wheat. Conversely, Ali et al. [11] found that Se application at 3 mg L^−1^ under drought stress conditions enhanced branch development in okra (*Abelmoschus esculentus* L.), suggesting a potential role of selenium in mitigating stress-induced growth limitations.

### 3.8. Chlorophyll a and Chlorophyll b and Total Chlorophyll

**Figure 2:**
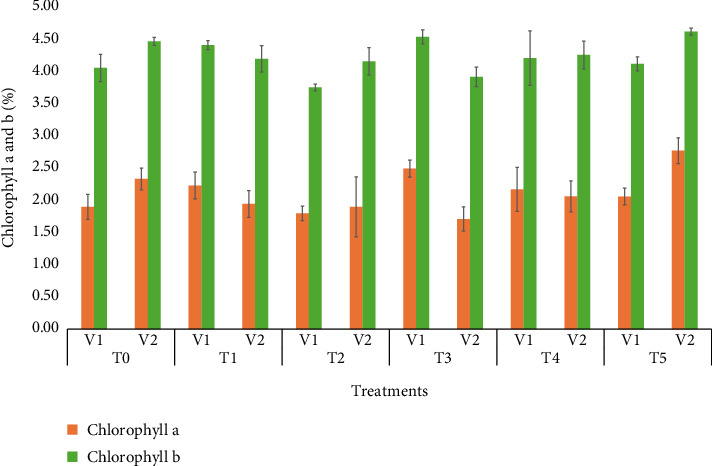
Means of chlorophyll a and chlorophyll b affected by wheat varieties and different selenium treatments.

Figure 2 demonstrates the best performance of chlorophyll a and b in the V_2_ variety (BARI Gom33), with chlorophyll a showing a content of 2.50% and chlorophyll b at 4.50%. Among the treatments, T_5_ (50 ppm Se) yielded the highest results, with 2.50% for chlorophyll a and 4.50% for chlorophyll b. The combination of T_5_ (50 ppm) × V_2_ (BARI Gom33) resulted in optimal performance, achieving a 4.55% increase in chlorophyll content. Previous studies have reported that the application of selenium helps protect chlorophyll structure and function from degradation, promoting an increase in photosynthetic pigment concentrations [34, 35]. Additionally, Liu et al. [36] found that foliar application of 4 mg L^−1^ Se enhanced chlorophyll a content in drought-tolerant cultivars, while both chlorophyll a and b contents were increased in drought-sensitive cultivars under heat stress conditions.

### 3.9. Carotenoid

**Figure 3:**
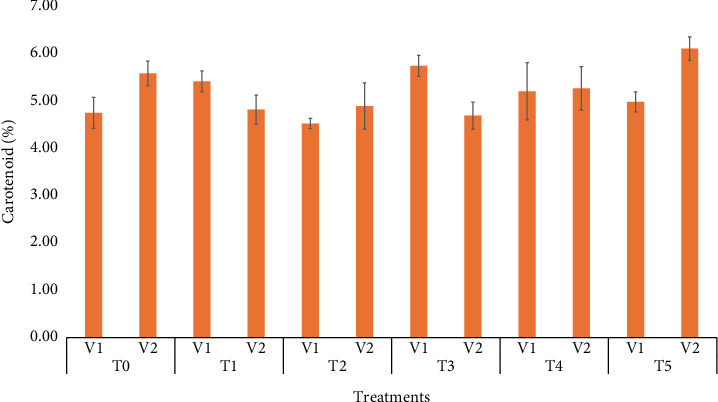
Means of carotenoid affected by wheat varieties and different selenium treatments.

Figure 3 illustrates that the highest carotenoid content (∼6.5%) was observed in V_2_ (BARI Gom33) with T_5_ (50 ppm), making it the most effective treatment. V_2_ consistently outperformed V_1_, demonstrating a better response to higher treatment levels. These results suggest that the combination of V_2_ (BARI Gom33) with T_5_ (50 ppm) is optimal for maximizing carotenoid accumulation. Silva et al. [37] highlighted that the enhancement of chlorophyll content plays a key role in better regulation of the antenna complex, which is associated with increased carotenoid content.

### 3.10. Relative Water Content and Water Saturation Deficit

**Figure 4:**
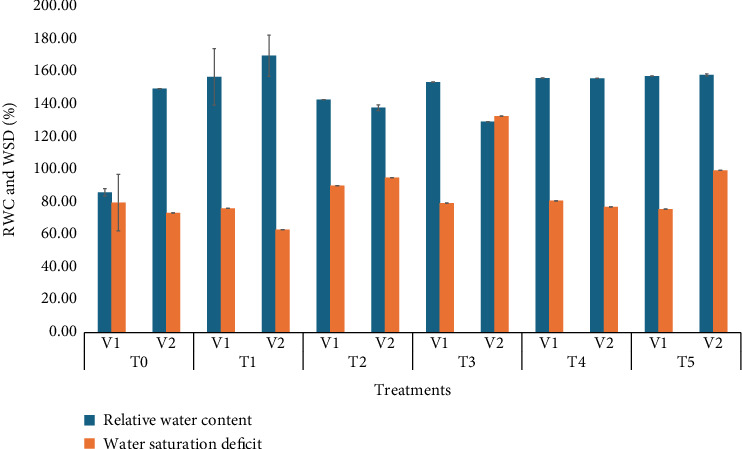
Means of RWC and WSD affected by wheat varieties and different selenium treatments.

The relative water content and water stress duration significantly varied with selenium application. The highest RWC (170%) was observed in V_1_ (BARI Gom30), as shown in Figure 4, while the highest WSD (130%) was observed in V_2_ (BARI Gom33). Regarding treatment effects, T_2_ (20 ppm) yielded the best result for RWC (170%), while the lowest RWC was recorded in T_0_. For WSD, the highest value was observed in T_0_, while the lowest was seen in T_2_. Iqbal et al. [38] suggested that SeNPs enhance plant properties, including RWC, under heat stress conditions. This improvement indicates that Se helps plants maintain better hydration, thereby mitigating the negative effects of stress and potentially enhancing stress tolerance and overall plant performance.

### 3.11. Water Retention Capacity (WRC) and Water Uptake Capacity

**Figure 5:**
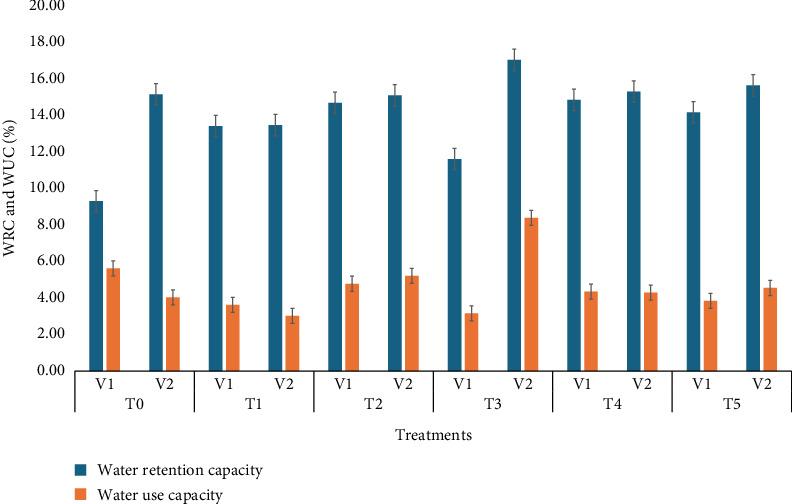
Means of water retention capacity (WRC) and water uptake capacity (WUC) affected by wheat varieties and different selenium treatments.

Figure 5 illustrates the effect of SeNPs on WRC and water uptake capacity in wheat varieties subjected to different selenium treatments. The results show that WRC is significantly enhanced by selenium treatments, with the 40 ppm dose yielding the highest retention. Notably, WRC consistently remained higher across all treatments compared to WUC, indicating that selenium primarily influences water retention rather than uptake. Furthermore, V_2_ varieties demonstrated higher WRC than V_1_, highlighting the role of genetic variation in response to selenium supplementation. This observation is consistent with findings from Iqbal et al. [38], which emphasize how SeNPs can improve stress tolerance and WUE in plants by enhancing their ability to retain water, particularly under drought conditions.

### 3.12. Leaf Succulence and ELWL

**Figure 6:**
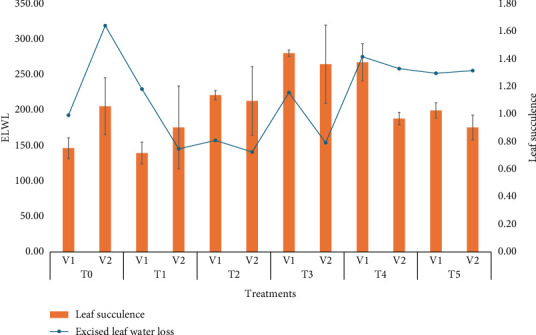
Means of leaf succulence and excised leaf water loss affected by wheat varieties and different selenium treatments.

The effects of treatments on leaf succulence and electrolyte leakage water loss revealed that increasing treatment concentrations initially reduce water loss, with the lowest ELWL observed at T_3_ (30 ppm), as shown in Figure 6. However, beyond T_3_, ELWL either stabilizes or slightly increases, suggesting that higher concentrations offer limited additional benefits. Leaf succulence fluctuates, with the highest values recorded at T_5_ (50 ppm), indicating improved water retention at moderate to higher concentrations. An inverse relationship exists between ELWL and leaf succulence, although it is not strictly linear. ELWL was most effective in V_1_ (BARI Gom30), with a value of 330%, while V_2_ (BARI Gom33) showed the lowest ELWL at 140%, making it particularly suited for water-limited conditions. Liu et al. [36] investigated the effects of SeNPs on two wheat varieties at different doses (10, 20, and 40 ppm). Their results demonstrated that SeNPs significantly enhanced leaf succulence and reduced ELWL, particularly at lower concentrations (10 and 20 ppm). At these doses, SeNPs improved the WRC of the leaves, leading to increased succulence.

### 3.13. CMS

**Figure 7:**
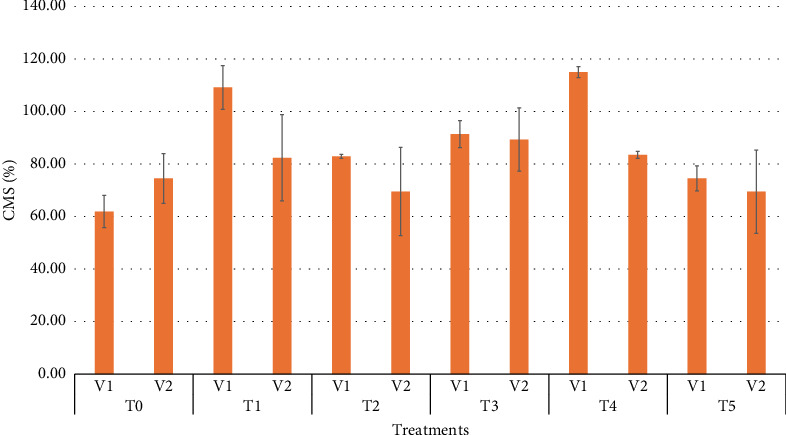
Means of cell membrane stability (CMS) affected by wheat varieties and different selenium treatments.

Figure 7 illustrates the effect of different treatments on the CMS across two wheat varieties. Notable variation in CMS is observed within the same variety, with some values exceeding 100%. Both V_1_ and V_2_ show a comparable range, though V_2_ (BARI Gom33) exhibited a slightly higher peak value. The CMS percentage varies across treatments, with T_3_ (30 ppm) consistently showing the highest CMS, suggesting it may be the most effective treatment. T_4_ (40 ppm) shows a decline compared to T_3_, indicating that higher concentrations may reduce effectiveness. T_0_ (control) and T_5_ (50 ppm) exhibited lower CMS values, suggesting that either no treatment or excessive concentrations negatively impact CMS. The treatment concentrations have a dominant effect on CMS, with T_3_ (30 ppm) emerging as the most effective dose, while concentrations exceeding 40 ppm may lead to reduced efficacy. Zhang et al. [39] reported that SeNPs at moderate doses, such as 30 ppm, enhance antioxidant defense systems, reduce oxidative stress, and stabilize cell membranes.

## 4. Conclusion

The results of the current study suggest that selenium supplementation as a nutritional element is beneficial under both normal and adverse environmental conditions. Se treatment effectively maintained various physiological and biochemical attributes by regulating LWP, relative water content, water saturation deficit, WRC, and water uptake capacity. Additionally, our findings indicated that the highest yield was achieved with treatment T_3_ (30 ppm). Se treatment also led to significant improvements in tiller number, leaf area, leaf number, spike length, chlorophyll a, and chlorophyll b content. Overall, V_2_ (BARI Gom33) exhibited slightly higher peak values across all morphological traits. However, further investigation into the individual and combined effects of Se treatment, as well as the response of different wheat varieties with and without Se under field conditions, is essential to validate the findings from controlled studies.

## Figures and Tables

**Table 1:** Effects of Se application on plant height, leaf number, tiller number, leaf area, leaf temperature, canopy temperature, spike length, biological yield, yield, and harvest index of wheat in different treatments.

Observation	Plant height (cm)	Leaf number	Tiller number	Leaf area (mm^2^)	Leaf temperature (°C)	Canopy temperature (°C)	Spike length (cm)	Biological yield (t ha^−1^)	Yield (t ha^−1^)	Harvest index
*Effect of variety*										
V_1_	97.41^b^	4.06^b^	3.056^b^	2651.00^a^	13.14	13.14	15.79^b^	3.59^b^	0.67^c^	35.28^b^
V_2_	102.63^a^	4.78^a^	3.78^a^	2649.00^b^	12.06	12.06	17.82^a^	3.83^a^	1.521^a^	40.00^a^

*Effect of Se nanoparticles*										
T_0_	67.07^c^	3.83^c^	2.50^c^	2133.65^d^	26.46	13.18	15.88^c^	3.26^c^	0.67^c^	29.89^b^
T_1_	99.67^c^	4.00^c^	2.12^c^	2582.23^cd^	26.51	12.95	16.73^ab^	3.38^cd^	1.00^b^	41.04^a^
T_2_	98.27^c^	5.00^bc^	2.20^c^	3087.26^bc^	26.48	11.48	16.85^ab^	3.70^bc^	1.55^a^	39.66^a^
T_3_	99.75^c^	4.33^c^	3.83^b^	3397.84^b^	26.53	12.45	16.92^ab^	3.86^b^	1.55^a^	40.02^a^
T_4_	101.43^b^	3.80^bc^	3.33^b^	3444.54^b^	26.52	13.30	16.72^bc^	3.71^bc^	1.53^a^	41.18^a^
T_5_	103.35^a^	5.50^a^	5.00^a^	5125.95^a^	26.53	12.23	17.75^a^	4.33^a^	1.47^a^	40.08^a^

*Interaction effect of variety and Se levels*										
T_0_ × V_1_	94.70^f^	3.33^e^	2.33^ef^	1890.19^e^	26.49	14.03	14.96^g^	2.96^f^	0.58^c^	19.88^d^
T_0_ × V_2_	100.63^c^	4.33^bcde^	2.67^def^	2377.11^cde^	26.43	12.33	16.8^cdef^	3.57^cde^	0.87^c^	39.90^abc^
T_1_ × V_1_	97.46^de^	4.67^bcd^	3.00^cde^	2320.93^de^	26.53	14.20	15.86^efg^	3.49^de^	1.38^b^	39.47^abc^
T_1_ × V_2_	101.86^c^	5.33^ab^	4.33^b^	2843.54^bcde^	26.49	11.70	17.60^bcd^	3.27^ef^	1.38^b^	42.59^a^
T_2_ × V_1_	95.60^ef^	3.67^de^	2.67^def^	3492.97^b^	26.50	11.90	15.80^efg^	3.73^bcde^	1.43^b^	38.41^abc^
T_2_ × V_2_	100.93^c^	4.33^bcde^	1.67^f^	3670.29^b^	26.50	11.06	17.90^bc^	3.66^bcdf^	1.50^b^	40.90^ab^
T_3_ × V_1_	97.10^de^	4.00^cde^	3.67^bcd^	3366.14^bc^	26.51	12.33	15.66^fg^	3.64^bcde^	1.50^b^	41.03^ab^
T_3_ × V_2_	102.40^bc^	4.67^bcd^	4.00^bc^	3429.54^b^	26.52	12.56	18.16^ab^	4.08^ab^	1.59^b^	39.00^abc^
T_4_ × V_1_	98.66^d^	3.67^de^	2.33^ef^	3218.78^bcd^	26.53	13.06	16.36^def^	3.61^bcde^	1.56^b^	39.94^abc^
T_4_ × V_2_	104.18^ab^	4.00^cde^	4.33^b^	3670.29^b^	26.52	13.53	17.06^bcde^	3.80^bcd^	2.00^a^	42.40^ab^
T_5_ × V_1_	100.93^c^	5.00^abc^	4.33^b^	4800.93^a^	26.54	13.30	16.10^efg^	4.07^bc^	2.11^a^	32.92^c^
T_5_ × V_2_	105.76^a^	6.00^a^	5.67^a^	5450.97^a^	26.51	11.16	19.40^a^	4.58^a^	2.23^a^	35.19^bc^

*F*-test	^∗∗^	^∗^	^∗∗^	^∗∗^	ns	ns	^∗∗^	^∗∗^	^∗∗^	^∗∗^
CV%	1.13	17.52	20.12	18.56	0.36	15.04	4.74	8.08	12.60	11.57

*Note:* V_1_: BARI Gom30, V_2_: BARI Gom33, T_0_: control, no selenium, T_1_: 10 ppm Se, T_2_: 20 ppm Se, T_3_: 30 ppm Se, T_4_: 40 ppm Se, and T_5_: 50 ppm Se. Different superscript letters indicate that groups have a statistically significant difference.

Abbreviation: ns, not significant.

^∗^,^∗∗^significant at *p* < 0.05 and 0.01, respectively.

## Data Availability

The data that support the findings of this study are available from the corresponding author upon reasonable request.
